# The structures of the kinase domain and UBA domain of MPK38 suggest the activation mechanism for kinase activity

**DOI:** 10.1107/S1399004713027806

**Published:** 2014-01-31

**Authors:** Yong-Soon Cho, Jiho Yoo, Soomin Park, Hyun-Soo Cho

**Affiliations:** a Department of Systems Biology, College of Life Science and Biotechnology, Yonsei University, Seoul 120-749, Republic of Korea

**Keywords:** MPK38, MELK, two-step activation model, UBA linker, activation-loop phosphorylation

## Abstract

Murine protein serine/threonine kinase 38 (MPK38) is the murine orthologue of human maternal embryonic leucine-zipper kinase (MELK), which belongs to the SNF1/AMPK family. MELK is considered to be a promising drug target for anticancer therapy because overexpression and hyperactivation of MELK is correlated with several human cancers. Activation of MPK38 requires the extended sequence (ExS) containing the ubiquitin-associated (UBA) linker and UBA domain and phosphorylation of the activation loop. However, the activation mechanism of MPK38 is unknown. This paper reports the crystal structure of MPK38 (T167E), which mimics a phosphorylated state of the activation loop, in complex with AMP-PNP. In the MPK38 structure, the UBA linker forces an inward movement of the αC helix. Phosphorylation of the activation loop then induces movement of the activation loop towards the C-lobe and results in interlobar cleft closure. These processes generate a fully active state of MPK38. This structure suggests that MPK38 has a similar molecular mechanism regulating activation as in other kinases of the SNF1/AMPK family.

## Abstract 2

The structure of a crystal of MPK38 (T167E), which consists of a kinase domain and a UBA domain, in complex with AMP-PNP is reported at 2.4 Å resolution. The structure indicates that the activation of MPK38 is induced by the UBA linker restraining the motion of the αC helix and by phosphorylation of Thr167 stabilizing the activation loop.

## 1. Introduction   

Murine protein serine/threonine kinase 38 (MPK38), also known as maternal embryonic leucine-zipper kinase (MELK), was initially identified as a murine orthologue of human HPK38/hMELK/KIAA175 (Gil *et al.*, 1997 ▶). MELK, the human orthologue of MPK38, is involved in the cell cycle (Davezac *et al.*, 2002 ▶), cell proliferation (Nakano *et al.*, 2005 ▶), apoptosis (Jung *et al.*, 2008 ▶; Lin *et al.*, 2007 ▶) and spliceosome assembly (Vulsteke *et al.*, 2004 ▶). Overexpression and hyperactivation of MELK are involved in initiation of the cell cycle but are barely detectable during cell differentiation (Badouel *et al.*, 2010 ▶). Recent research shows that MELK expression is correlated with malignancy in a broad spectrum of cancers (Gray *et al.*, 2005 ▶; Marie *et al.*, 2008 ▶; Nakano *et al.*, 2008 ▶; Pickard *et al.*, 2009 ▶; Ku *et al.*, 2010 ▶). Overexpression of MELK is associated with a poor prognosis in breast cancer and glioblastoma patients. MELK is hyperactivated in cell lines derived from a colorectal carcinoma (Pickard *et al.*, 2009 ▶; Nakano *et al.*, 2008 ▶; Ku *et al.*, 2010 ▶). MELK plays a critical role in the formation or maintenance of cancer stem cells (Sutter *et al.*, 2007 ▶; Hebbard *et al.*, 2010 ▶). Therefore, MELK is recognized as an emerging target for anticancer drug therapy.

MPK38 is a serine/threonine kinase that belongs to the SNF1/AMPK family, which contains yeast sucrose-non-fermenting-1 (Snf1), two isoforms of AMP-activating protein kinase (AMPKα1 and AMPKα2) and 12 AMPK-related kinases (Beullens *et al.*, 2005 ▶; Bright *et al.*, 2009 ▶; Manning *et al.*, 2002 ▶). All kinases in this family have a conserved threonine residue (Thr172 in AMPKα1 and AMPKα2 and Thr167 in MPK38) in their activation loop (Beullens *et al.*, 2005 ▶; Bright *et al.*, 2009 ▶; Woods *et al.*, 2003 ▶). Phosphorylation of the threonine residue in the activation loop of AMPKα1 and AMPKα2 is triggered by the upstream kinase LKB1, while phosphorylation of the activation loop of MPK38 occurs by autophos­phorylation (Bright *et al.*, 2009 ▶; Lizcano *et al.*, 2004 ▶; Woods *et al.*, 2003 ▶). Substitution of Thr with a negative charged amino acid in the activation loop of MPK38 induces constitutive activation (Beullens *et al.*, 2005 ▶).

MPK38 consecutively comprises a kinase domain (KD), a ubiquitin-associated (UBA) domain, a TP dipeptide-rich domain and a kinase-associated 1 (KA1) domain. A C-terminal fragment of MPK38 containing the TP dipeptide-rich domain and the KA1 domain functions as an autoinhibitory domain. Ten AMPK-related kinases, including MPK38, contain a UBA domain, which is a specific feature that only occurs in the mammalian genome (Bright *et al.*, 2009 ▶; Jaleel *et al.*, 2006 ▶). The UBA domain binds to polyubiquitinated proteins, which inhibits further ubiquitination and attenuates proteosomal degradation of the target protein (Buchberger, 2002 ▶; Davies *et al.*, 2004 ▶; Hartmann-Petersen *et al.*, 2003 ▶; Mueller *et al.*, 2004 ▶). The UBA domain is also involved in protein–protein interactions such as protein dimerization (Bertolaet *et al.*, 2001 ▶). The extended sequence (ExS) containing the UBA linker is involved in regulation of activity in AMPK-related kinases (Beullens *et al.*, 2005 ▶; Jaleel *et al.*, 2006 ▶). The kinase domain (KD) and the UBA domain are connected by the UBA linker, which has a length of approximately 20 amino acids. The ExS consists of the UBA linker and UBA domain.

The activity of protein kinases depends on the correct positioning of the active-site residues (Huse & Kuriyan, 2002 ▶; Nolen *et al.*, 2004 ▶). Most kinases are activated by phosphorylation of a Ser/Thr or a Tyr residue in the activation loop (Canagarajah *et al.*, 1997 ▶; Krupa *et al.*, 2004 ▶). However, some kinases have an alternative activation mechanism, such as those observed in the platelet-derived growth factor receptor (PDGFR), cyclin-dependent kinase (CDK) and AGC kinase families. The juxtamembrane domain of the PDGFR family is released from the KD for activation (Griffith *et al.*, 2004 ▶; Irusta *et al.*, 2002 ▶). Intermolecular interactions are formed between CDKs and cyclins before phosphorylation of CDKs (Jeffrey *et al.*, 1995 ▶; Johnson & O’Reilly, 1996 ▶; Pavletich, 1999 ▶; Russo *et al.*, 1996 ▶). In the AGC family, intramolecular interactions between the KD and the C-terminal flanking sequence are required for activation (Gold *et al.*, 2006 ▶; Knighton *et al.*, 1991 ▶; Yang, Cron, Good *et al.*, 2002 ▶; Yang, Cron, Thompson *et al.*, 2002 ▶).

The activation mechanism of UBA-containing kinases has not yet been identified. Previous work indicated that MPK38 was not activated without the UBA linker and the UBA domain (Beullens *et al.*, 2005 ▶). Other AMPK-related kinases containing ExS show a similar activation mechanism to that of MPK38 (Jaleel *et al.*, 2006 ▶). The structures of UBA-containing kinases such as MARK1, MARK2 and MARK3 have been determined (Marx *et al.*, 2006 ▶; Murphy *et al.*, 2007 ▶; Nešić *et al.*, 2010 ▶; Panneerselvam *et al.*, 2006 ▶). Although an active conformation of MARK2 has been reported in the presence of an inhibitory peptide from *Helicobacter pylori*, there is no active structure in the absence of peptide. Therefore, the structures do not disclose the structural role of the factors that induce or stabilize the active conformation in the absence of peptide (such as the UBA linker, the UBA domain and activation loop phosphorylation; Marx *et al.*, 2006 ▶; Murphy *et al.*, 2007 ▶; Nešić *et al.*, 2010 ▶; Panneerselvam *et al.*, 2006 ▶).

This study identified the activation mechanism of MPK38. We determined the structure of MPK38 (T167E) including the KD and the ExS, which mimics the activated state of MPK38. Our results revealed that the positioning of residues in the active site was induced by the UBA linker in the ExS, which restrained the movement of the αC helix. Additionally, the phosphorylation-mimic mutation in the activation loop triggered reorganization of the activation loop and the interlobar cleft into a more closed conformation. The structure of MPK38 (T167E) suggests that there is a common activation mechanism among kinases of the SNF1/AMPK family.

## 2. Materials and methods   

### 2.1. Protein expression and purification   

Residues 1–326 of MPK38 were subcloned into the pET-21b vector (Novagen). The MPK38 (T167E) mutant, which mimics a constitutively phosphorylated state, was generated using the QuikChange kit (Stratagene). The MPK38 (T167E) construct was transformed into *Escherichia coli* BL21(DE3) cells. The expression of MPK38 (T167E) was induced by 0.5 m*M* IPTG at 18°C for 16 h. The cells were harvested and suspended in cell-lysis buffer (20 m*M* Tris–HCl pH 7.5, 500 m*M* NaCl). The cells were lysed by sonication and the supernatant was separated by centrifugation. The cell supernatant was applied onto a HisTrap HP column (GE Healthcare) and washed with washing buffer (20 m*M* Tris–HCl pH 7.5, 500 m*M* NaCl, 50 m*M* imidazole). MPK38 (T167E) was eluted with elution buffer (20 m*M* Tris–HCl pH 7.5, 500 m*M* NaCl, 200 m*M* imidazole). MPK38 (T167E) was purified using a HiTrap Q anion-exchange column (GE Healthcare). Finally, MPK38 (T167E) was eluted by gel-filtration chromatography (HiLoad 16/60 200 pg, GE Healthcare) in size-exclusion chromatography (SEC) buffer (20 m*M* Tris–HCl pH 7.5, 300 m*M* NaCl, 5 m*M* DTT).

### 2.2. Crystallization, data collection and structure determination   

MPK38 (T167E) was concentrated to 15 mg ml^−1^ for crystallization screening using a commercial screening kit (Hampton Research). MPK38 (T167E) was mixed with 1 m*M* AMP-PNP and a complex crystal was grown at 293 K by the vapour-diffusion method using 1.7 *M* ammonium sulfate, 50 m*M* CAPSO pH 9.8 in two weeks. The complex crystal was soaked in crystallization buffer with 20% ethylene glycol before cooling. X-­ray diffraction data were collected from the crystals on the BL-17A beamline at the Photon Factory, Japan. The MPK38 (T167E) crystal belonged to space group *P*2_1_2_1_2_1_, with unit-cell parameters *a* = 35.799, *b* = 75.711, *c* = 128.088 Å. The initial phase of MPK38 (T167E) was obtained by molecular replacement using *MOLREP* (Vagin & Teplyakov, 2010 ▶) with the human MELK structure (PDB entry 3zgw; G. Canevari, S. Re-Depaolini, U. Cucchi, B. Forte, P. Carpinelli & J. A. Bertrand, unpublished work) as a search model. The model of MPK38 (T167E) was built using *Coot* (Emsley *et al.*, 2010 ▶), and *PHENIX* was used for structure refinement (Adams *et al.*, 2010 ▶). The statistics of data collection and refinement are presented in Table 1 ▶.

## 3. Results and discussion   

### 3.1. Overall structure of the MPK38 constitutively active mutant   

**Figure 1:**
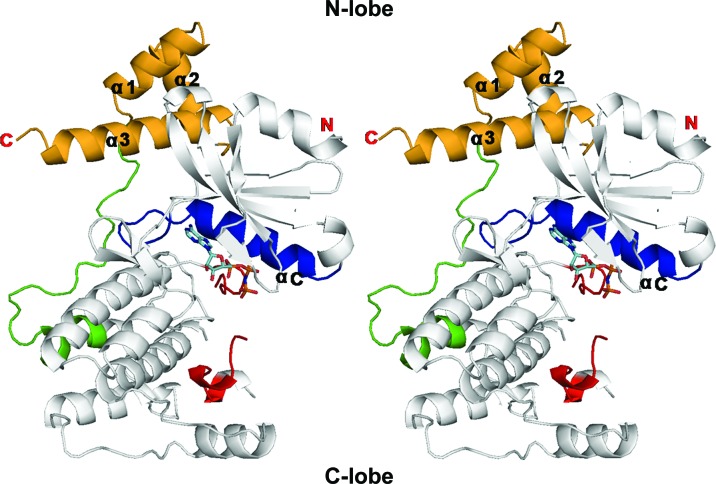
Overview of the structure of MPK38 (T167E): stereo image of the MPK38 (T167E) structure. The UBA domain is shown in yellow, the UBA linker in green, the activation loop in red, the αC helix in blue and AMP-PNP in cyan.

**Figure 2:**
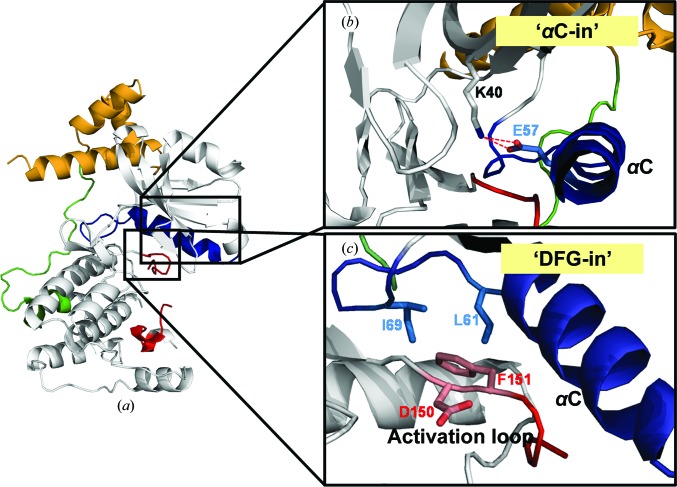
Conformation of the active-state kinase domain (KD) in MPK38 (T167E). The UBA domain is shown in yellow, the UBA linker in green, the activation loop in red and the αC helix in blue. (*a*) Overview of the MPK38 (T167E) structure. (*b*) Close-up view of the αC-in conformation. The ion pair between Lys40 and Glu57 is marked as a red dashed line. (*c*) Close-up view of the DFG-in conformation.

The overall structure of MPK38 (T167E) shows the KD and ExS, which contain the UBA linker and the UBA domain (Beullens *et al.*, 2005 ▶). In MPK38, Thr167 is substituted by Glu to mimic the phosphorylated state of the activation loop. The KD of MPK38 (T167E) adopts the classical bilobar protein kinase fold, which consists of an N-terminal lobe (N-lobe; residues 1–91) and a C-terminal lobe (C-lobe; residues 94–263) (Huse & Kuriyan, 2002 ▶; Knighton *et al.*, 1991 ▶; Nolen *et al.*, 2004 ▶; Fig. 1 ▶). The ATP-binding pocket is located between the N- and C-lobes. The N-­lobe, which contains six antiparallel β-sheets and the regulatory αC helix, is involved in ATP binding and in inter­action with the UBA domain (residues 284–326). The C-lobe consists predominantly of α-­helices and serves as a docking site for substrates. It includes the catalytic loop (residues 132–137), the DFG motif (the magnesium-binding loop) and the activation loop (residues 150–176) that participates in the transfer of phosphate to substrates. The N-­lobe exhibits the αC-in and DFG-in conformations, which are conserved throughout all active protein kinases (Figs. 2 ▶ *b* and 2 ▶ *c*). The activation loop is localized in the C-lobe of the KD (Fig. 2 ▶ *d*), although 11 residues (residues 158–168) in the activation loop are not visible in the electron-density map. The AMP-PNP, which is an analogue of ATP, is positioned in the ATP-binding pocket (Fig. 1 ▶).

**Figure 3:**
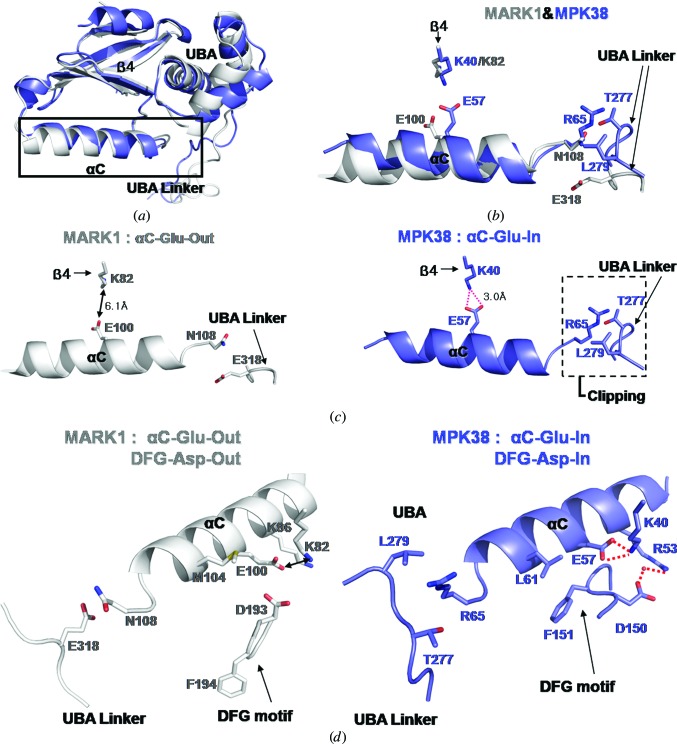
Activation of MPK38 by the UBA linker. (*a*) Superposition of the MPK38 (T167E) N-lobe (blue) and the MARK1 N-lobe (white). (*b*, *c*) Detailed interactions are shown among the KD αC helix, KD β4 and the UBA linker. The key residues that show conformational differences in the KDs of MPK38 and MARK1 are labelled. The distance between Glu in αC and Lys in β4 is shown. (*d*) Structures of MARK1 (left, white) and MPK38 (T167E) (right, blue) presented in different views to those in (*b*). The distance between Glu in αC and Lys in β4 is shown for both proteins (6.1 Å in MARK1 and 3.0 Å in MPK38). The ion pairs are displayed as red dashed lines.

The UBA domain of MPK38 is a small globular domain that contains three short helices (α1–α3) and binds to the back of the N-lobe (Fig. 1 ▶). Helices α1 and α3 are approximately antiparallel in conformation, as observed for those in the MARKs (MARK1, MARK2 and MARK3; Marx *et al.*, 2006 ▶; Murphy *et al.*, 2007 ▶; Panneerselvam *et al.*, 2006 ▶). The UBA domain is linked to the KD by the UBA linker residues 264–­283. The UBA domain is conserved among UBA-containing kinases. The UBA domain of MPK38 shows a similar conformation as in the MARKs. The UBA linker is not conserved; the MPK38 linker exhibits a unique conformation unlike that in the MARKs (Fig. 3 ▶ and Supporting Fig. S1^1^).

### 3.2. Structural comparison between MPK38 and MARK1   

#### 3.2.1. The role of ExS in MPK38   

Protein kinases are generally activated by phosphorylation of their activation loop. However, a few kinases are activated by alternative mechanisms. The AGC-family kinases are a typical example, in which the C-terminal hydrophobic motif is required for protein stability and kinase activity (Gold *et al.*, 2006 ▶). Similarly, the activation of MPK38 requires phosphorylation of the activation loop and the C-terminal ExS (Beullens *et al.*, 2005 ▶; Jaleel *et al.*, 2006 ▶). The structures of three UBA-containing kinases that contain an ExS, including MARK1, MARK2 and MARK3, have been determined in the inactive state (Marx *et al.*, 2006 ▶; Murphy *et al.*, 2007 ▶; Nešić *et al.*, 2010 ▶; Panneerselvam *et al.*, 2006 ▶). The structural conformation of MPK38 (T167E) is quite different from the structures of the three MARKs. The MARK structures display the canonical inactive αC-out, DFG-out conformation and a severely disordered activation loop (Marx *et al.*, 2006 ▶; Murphy *et al.*, 2007 ▶; Panneerselvam *et al.*, 2006 ▶). Our MPK38 (T167E) structure reflects an active conformation with an αC-in, DFG-­in conformation and the activation loop localized at the C-­lobe (Figs. 2 ▶ *b*, 2 ▶ *c* and 4 ▶ *a*). A comparison of the MPK38 (T167E) and MARK structures reveals that the ExS affects the KD conformation and indicates the conformational changes that occur during activation of the UBA-containing kinases. The MARKs have ∼90% sequence identity and their structures are conserved; therefore, the human MARK1 structure was used in comparative studies with MPK38 (T167E).

The structures of MPK38 and MARK1 were separately aligned for the N-lobe and the C-lobe, with r.m.s.d.s of 0.58 and 0.82 Å, respectively. In the N-lobe, there is an obvious difference at the end of αC in the hinge region (Figs. 3 ▶ *a* and 3 ▶ *b*). In MPK38, the UBA linker and the N-lobe make extensive contacts through strong van der Waals interactions and a hydrogen bond (Supporting Figs. S2 and S3). In contrast, the UBA linker and the N-lobe are far apart at the end of αC in MARK1, so there is only one van der Waals contact (Figs. 3 ▶ *b* and 3 ▶ *c*). This unambiguous structural difference clearly shows that rearrangement of the UBA linker causes a conformational change of the KD N-lobe from an inactive to an active state. In MPK38, Thr277 and Leu279 of the UBA linker modulate the αC helix from the ‘out’ to the ‘in’ position *via* a strong van der Waals interaction with Arg65 of the αC helix and an additional hydrogen bond in the vicinity (Supporting Figs. S2 and S3); in detail, the O atom of the hydroxyl group of Thr277 interacts with the C^γ^ and N^∊^ atoms of Arg65 at distances of 3.4 and 3.6 Å, respectively. The C^δ^ atom of Leu279 makes contacts with the N^η^ and C atoms of the guanidinium moiety of Arg65 at distances of 3.5–3.6 Å. In MARK1, Glu318 of the UBA linker keeps the αC helix in the ‘out’ position *via* a relatively weak van der Waals interaction with Asn108 (4.0 Å; Fig. 3 ▶ *c*). Thus, extensive hydrophobic inter­actions in MPK38 between Arg65 in KD and Leu279 in the UBA linker function as a clip that restrains the motion of the regulatory αC helix (Fig. 3 ▶ *c*). This arrangement leads to the following interactions: Glu57 in the αC helix engages in favourable electrostatic interactions with Lys40 (Fig. 3 ▶ *c*), while hydrophobic interactions between Leu61 and Phe151 in the DFG motif and a water-mediated hydrogen bond between Arg53 in the αC helix and Asp150 of the DFG motif induce the DFG-in conformation (Fig. 3 ▶ *d*). Thr277 and Leu279, which are the key residues for the clipping function of the UBA linker, are fixed in position by the following interactions. Firstly, the C-­terminal residue, Leu279, is stabilized by van der Waals contacts with Trp308 from the UBA domain (Supporting Fig. S4*a*). Secondly, the N-­terminal residue, Ser275, is located in the vicinity of the αC helix by a hydrogen bond to the main-chain O atom of Arg65 (Supporting Fig. S4*b*). Thirdly, most of the N-terminal residues are fixed at the C-lobe of KD by multiple van der Waals interactions. In detail, Trp262, Val263, Tyr267, Val271 and Trp273 of the UBA linker pack against the C-lobe surface lined by Arg110, Val111, Arg114, Leu117, Ser118 and Ser125 (Supporting Fig. S4*c*). Additionally, the recently deposited human MELK structure (PDB entry 3zgw; G. Canevari, S. Re-Depaolini, U. Cucchi, B. Forte, P. Carpinelli & J. A. Bertrand, unpublished work) is very similar to that of MPK38, with an r.m.s.d. of 0.453 Å over 253 amino-acid residues. The αC-in and DFG-in conformation in KD and the interactions between the UBA linker and αC are found in both structures. In particular, although the key residues in the UBA linker are different (the corresponding residues to Thr277 and Leu279 in MPK38 are Asn277 and Phe279 in human MELK), the UBA linker also restrains αC *via* strong van der Waals inter­actions in the human enzyme, showing the conserved role of the UBA linker. In detail, the O atom of the side chain of Asn277 interacts with the N^∊^ atom of Arg65 at a distance of 3.8 Å. The phenyl ring of Phe279 makes a contact with the N^∊^ and C^δ^ atoms of Arg65 at a distance of 3.5–3.6 Å. Moreover, the additional hydrogen bonding between the carbonyl group of the main chain of Arg65 and the hydroxyl group of Ser275 is also conserved (Supporting Fig. S5). This function of the UBA linker is reminiscent of cyclins, which interact with and activate their cognate CDKs. Cyclins also restrain the movement of the αC helix, which promotes the interaction between a Glu in the αC helix and a Lys in the N-lobe. This interaction holds the phosphate of ATP in the correct position (Pavletich, 1999 ▶).

#### 3.2.2. The role of phosphorylated Thr167 in MPK38   

**Figure 4:**
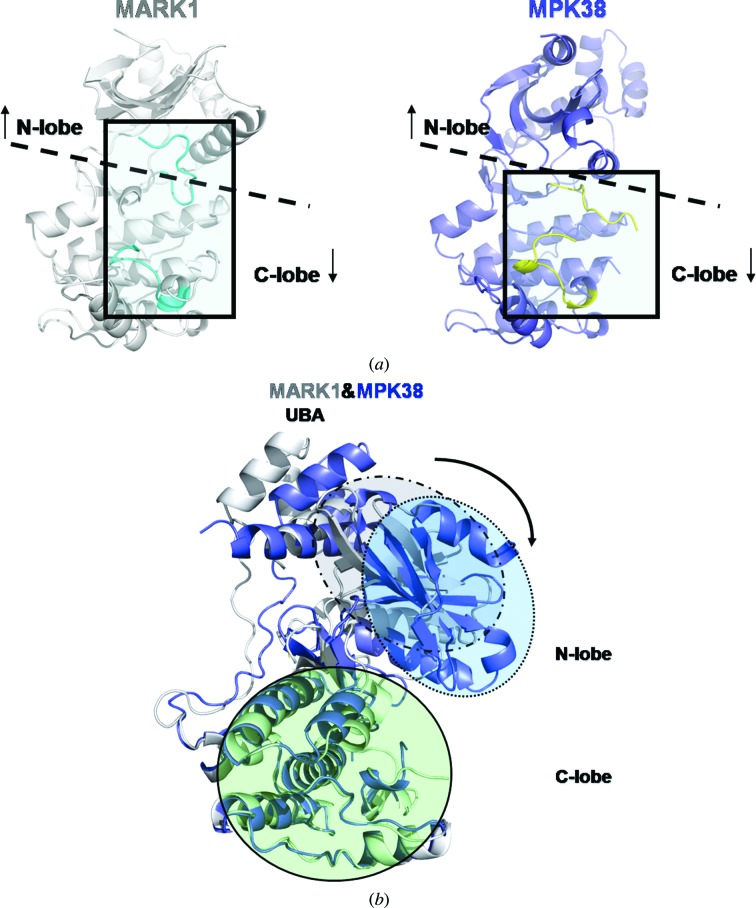
Activation of MPK38 by phosphorylation of the activation loop. (*a*) The activation loop of MARK1 (white) and MPK38 (T167E) (blue) are shown as boxed regions. (*b*) The interlobar cleft in MARK1 has a more open conformation compared with that of MPK38 (T167E). MARK1 (white) and MPK38 (T167E) (blue) are superposed and the N-lobes and C-lobes of their KDs are highlighted by ellipses. The N-lobe of MARK1 is shown in the grey ellipse, the N-lobe of MPK38 (T167E) is shown in the blue ellipse and the C-lobes of both proteins are shown in the green ellipse.

The MPK38 and MARK1 structures show significant differences in the conformational change of the activation loop and the opening/closing of the interlobar cleft. The substitution of Thr167 by Glu in MPK38 causes the activation loop to move towards the C-lobe of KD compared with that in MARK1, in which the activation loop moves towards the N-lobe (Fig. 4 ▶ *a*). Although our structure of MPK38 does not identify 11 residues in the activation loop, including Glu167 that mimics the structure of phosphorylated Thr167, it shows that the activation loop is relatively stabilized in the C-lobe (Fig. 4 ▶ *a*). The activation loop in our MPK38 structure is partially stabilized because the glutamate does not completely mimic a phosphorylated state and the interactions with the surrounding residues (such as Arg131 of the catalytic loop) are not stable. This observation is consistent with a previous study of a MELK mutant that had a negatively charged amino acid substituted for the activation-loop Thr; this mutant appeared to have a lower activity than that of the autophosphorylated wild-type kinase (Beullens *et al.*, 2005 ▶). The conformational shift of the activation loop decreases the restriction of the rotation of the N-­lobe towards the C-­lobe, thus inducing the interlobar cleft to move from the open to the relatively closed conformation (Fig. 4 ▶ *b*).

### 3.3. Structural insights into regulation of the activity of MPK38 and the SNF1/AMPK family   

**Figure 5:**
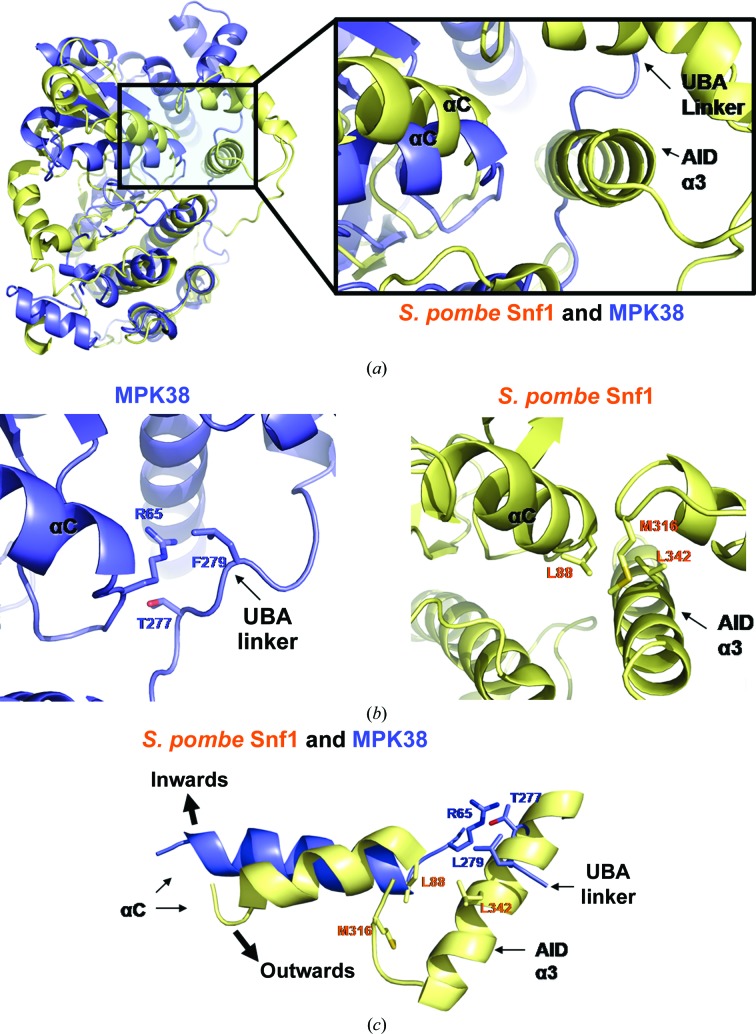
Comparison of the activation of MPK38 by ExS and the inhibition of Snf1 (from *S. pombe*) by AID. (*a*) Structural comparison of MPK38 (T167E) (blue) with Snf1 (yellow) is shown *via* an overall view (left) and a close-up view (right) of the boxed region. (*b*) Each protein in the boxed region of (*a*) is displayed separately. The structures show the van der Waals contacts at the interface of the KD–UBA linker region of MPK38 and those at the KD–AID interface of Snf1. (*c*) A close-up view of the region around the αC helix in both proteins shows that the interactions observed in (*b*) force αC to rotate inwards (MPK38) or outwards (Snf1).

The SNF1/AMPK family contains yeast Snf1, AMPKα1, AMPKα2 and 12 AMPK-related kinases including ten UBA-containing kinases (Bright *et al.*, 2009 ▶; Manning *et al.*, 2002 ▶). The SNF1/AMPK-family KDs are conserved; however, there is significant divergence among the sequences that encode the remaining regions of the proteins (Bright *et al.*, 2009 ▶). Structural studies of Snf1, which is the yeast orthologue of mammalian AMPK, report that the autoinhibition of AMPKs is regulated by interactions between the KD and an autoinhibitory domain (AID; Chen *et al.*, 2009 ▶). Our structural study of MPK38 reveals that activation occurs *via* interactions between the KD and the ExS that involve the UBA linker. These interactions commonly restrain the motion of the αC helix and influence kinase activity in both AMPK and MPK38. Although the Snf1 AID binds to the KD using a similar mechanism as that for the binding of the MPK38 UBA linker to the KD, its function in the regulation of activity is different (Chen *et al.*, 2009 ▶). In *Schizosaccharomyces pombe* Snf1, Leu88 at the end of αC interacts with Met316 on α1 and Leu342 in the AID of α3 (Figs. 5 ▶ *a* and 5 ▶ *b*; Chen *et al.*, 2009 ▶). In MPK38, Arg65 at the end of αC interacts with Thr277 and Leu279 in the UBA linker (Figs. 5 ▶ *a* and 5 ▶ *b*). AID binding forces the αC helix outwards and inhibits kinase activity, whereas the UBA linker forces the αC helix inwards and induces kinase activation (Fig. 5 ▶ *c*; Chen *et al.*, 2009 ▶). Taken together, MPK38 and AMPKs regulate the motion of the αC helix *via* similar binding-mode mechanisms, in which the UBA linker and the AID interact with the hinge region but show completely opposite effects (activation and inhibition, respectively). These effects are owing to different restraints that determine the direction of movement of the αC helix.

## Supplementary Material

PDB reference: MPK38 (T167E), complex with AMP-PNP, 4bfm

Supporting information file. DOI: 10.1107/S1399004713027806/cb5036sup1.pdf

## Figures and Tables

**Table 1:** X-ray data-collection and refinement statistics Values in parentheses are for the highest resolution shell.

Data collection	
Space group	*P*2_1_2_1_2_1_
Unit-cell parameters (Å, °)	*a* = 35.759, *b* = 75.711, *c* = 128.088, α = β = γ = 90
Resolution (Å)	50–2.35
*R*_merge_	0.101 (0.355)
Average *I*/σ(*I*)	35.78 (6.21)
Completeness (%)	99.8 (99.1)
Multiplicity	10.8 (7.0)
Refinement	
Resolution (Å)	48.90–2.35
No. of reflections	15156
*R*_work_/*R*_free_	0.17/0.20
No. of atoms	
Protein	2267
Water	21
*B* factors (Å^2^)	
Protein	93.52
Water	86.40
R.m.s. deviations	
Bond lengths (Å)	0.012
Bond angles (°)	1.242

## Footnotes

1 Supporting information has been deposited in the IUCr electronic archive (Reference: CB5036).
