# Supplementary material for: The structures of the kinase domain and UBA domain of MPK38 suggest the activation mechanism for kinase activity
Source: Acta Crystallogr D Biol Crystallogr. 2014 Jan 31;70(Pt 2):514–21. doi: 10.1107/S1399004713027806 (PMC3940201; doi:10.1107/S1399004713027806)
Supplement: Supplementary file 1 [file d-70-00514-sup1.pdf]

Supporting Information

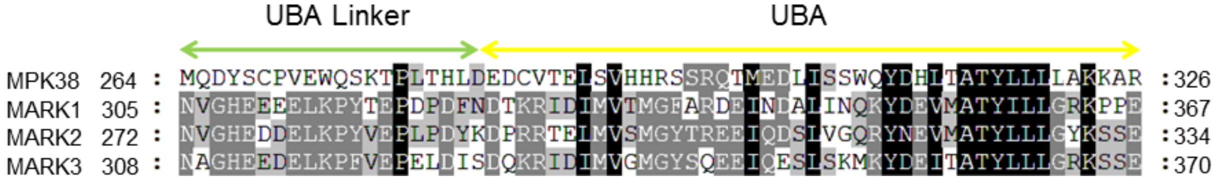

Supporting Figure S1 Multiple sequence alignments of ExS spanning the UBA linker and the UBA domain) of MPK38, MARK1, MARK2, and MARK3. The alignment was made with ClustalW (Higgins D et al., 1994) and shaded with Genedoc (www.nrbsc.org/gfx/genedoc/index.html).

(a)

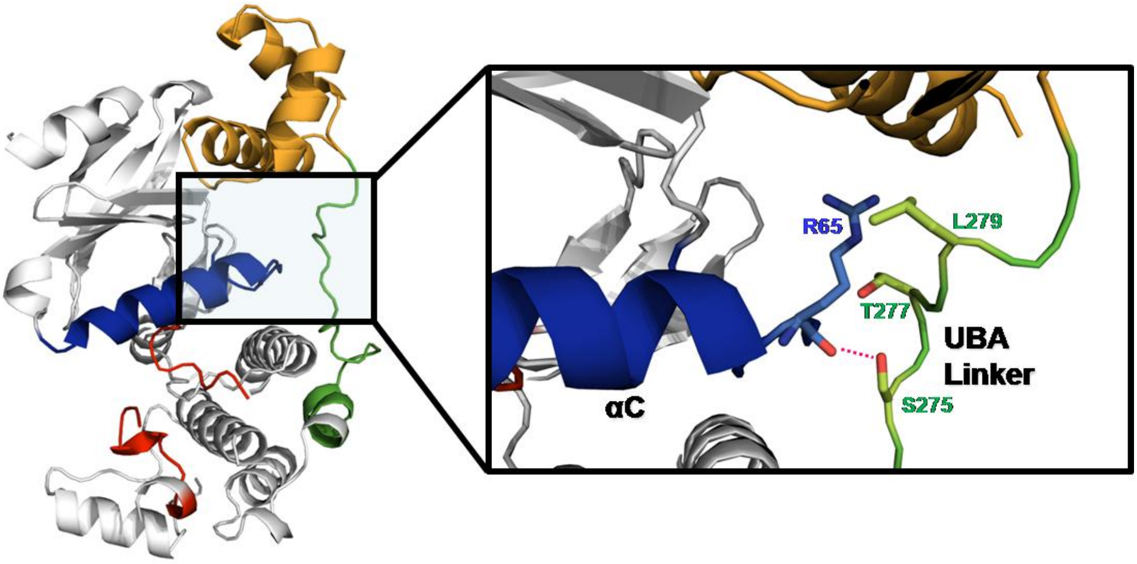

(b)

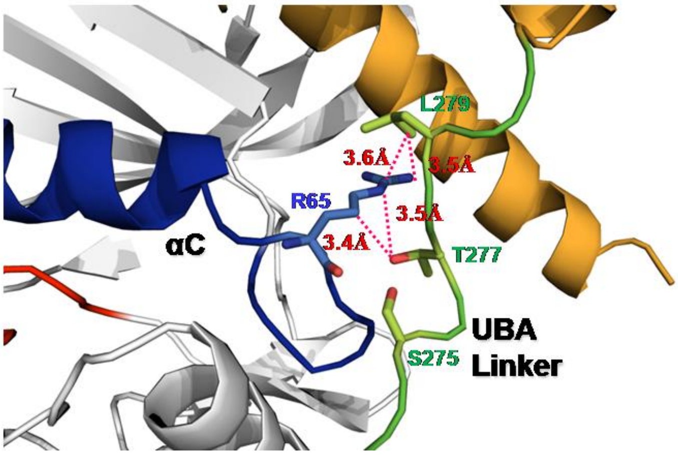

Supporting Figure S2 Interaction between the UBA linker and the N-lobe. (a) The overall structure of MPK38 (T167E) and a close-up view of the boxed region are shown. The hydrogen bond is displayed as red dashed lines. (b) The atomic interactions between Thr277/Leu279 and Arg65 are shown as red dashed lines. The distances of the specific interactions are indicated. The region are shown as UBA domain (yellow), UBA linker (green), activation loop (red), and αC helix (blue).

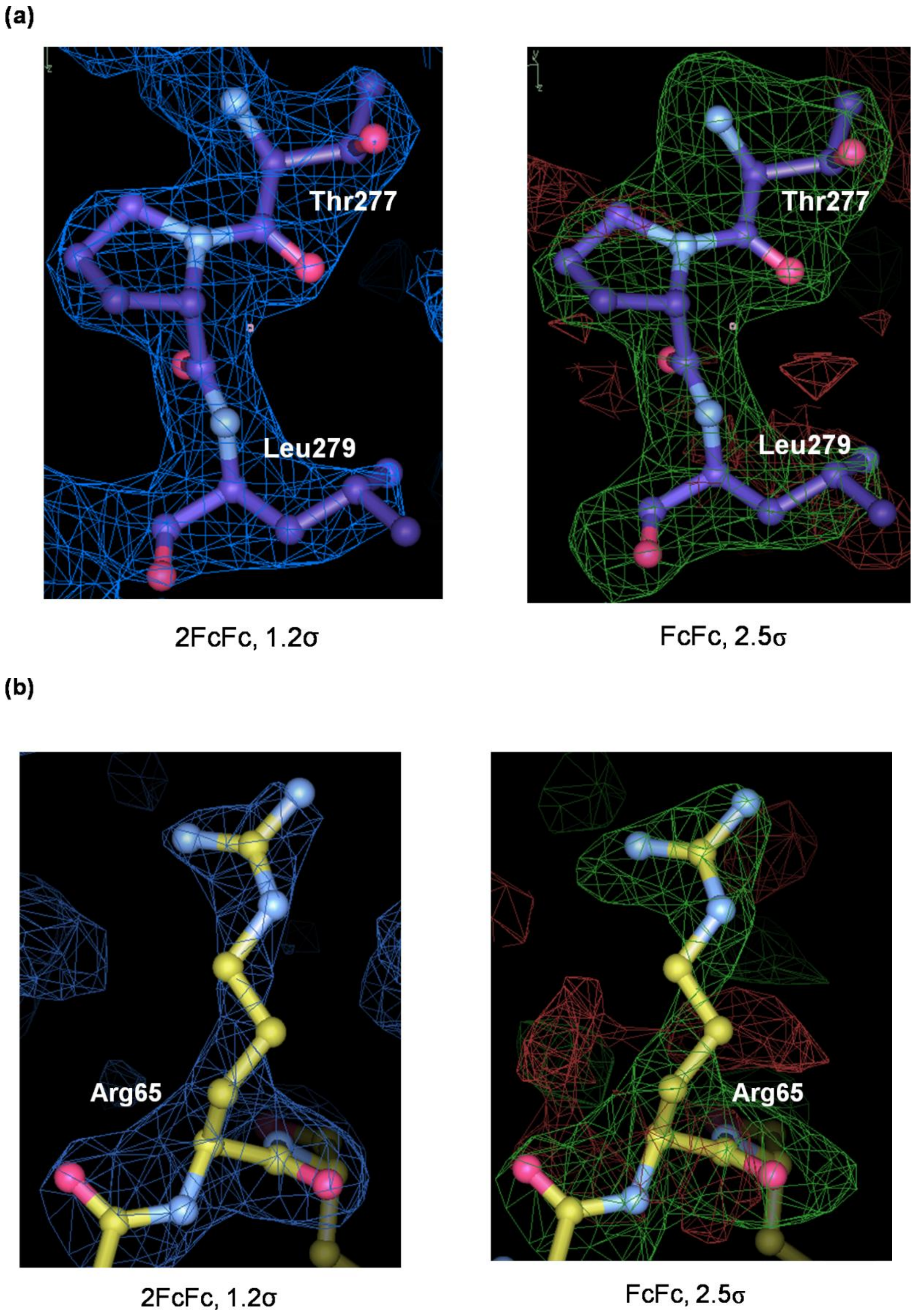

Supporting Figure S3 Electron density map for Thr277, Leu279 and Arg65. (a) 2FoFc (left) and FoFc (right) electron density map for Thr277/Leu279 are shown respectively. (b) 2FoFc (left) and FoFc (right) electron density map for Arg65 are shown.

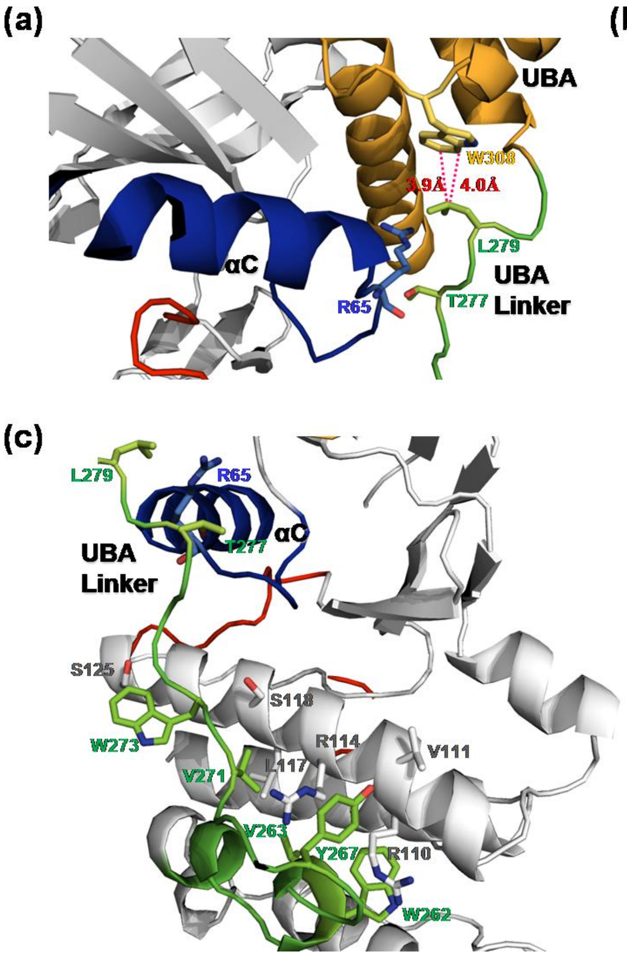

Supporting Figure S4 The interactions locating at the current position of Thr277 and Leu279. (a) The atomic interactions between Leu279 and Trp308 are shown as red dashed lines. The distances of the specific interactions are indicated. (b) The hydrogen bond between Arg65 and Ser275 is displayed as red dashed lines. (c) The residues participating in the interactions between the UBA Linker and the C-lobe of KD are shown as stick.

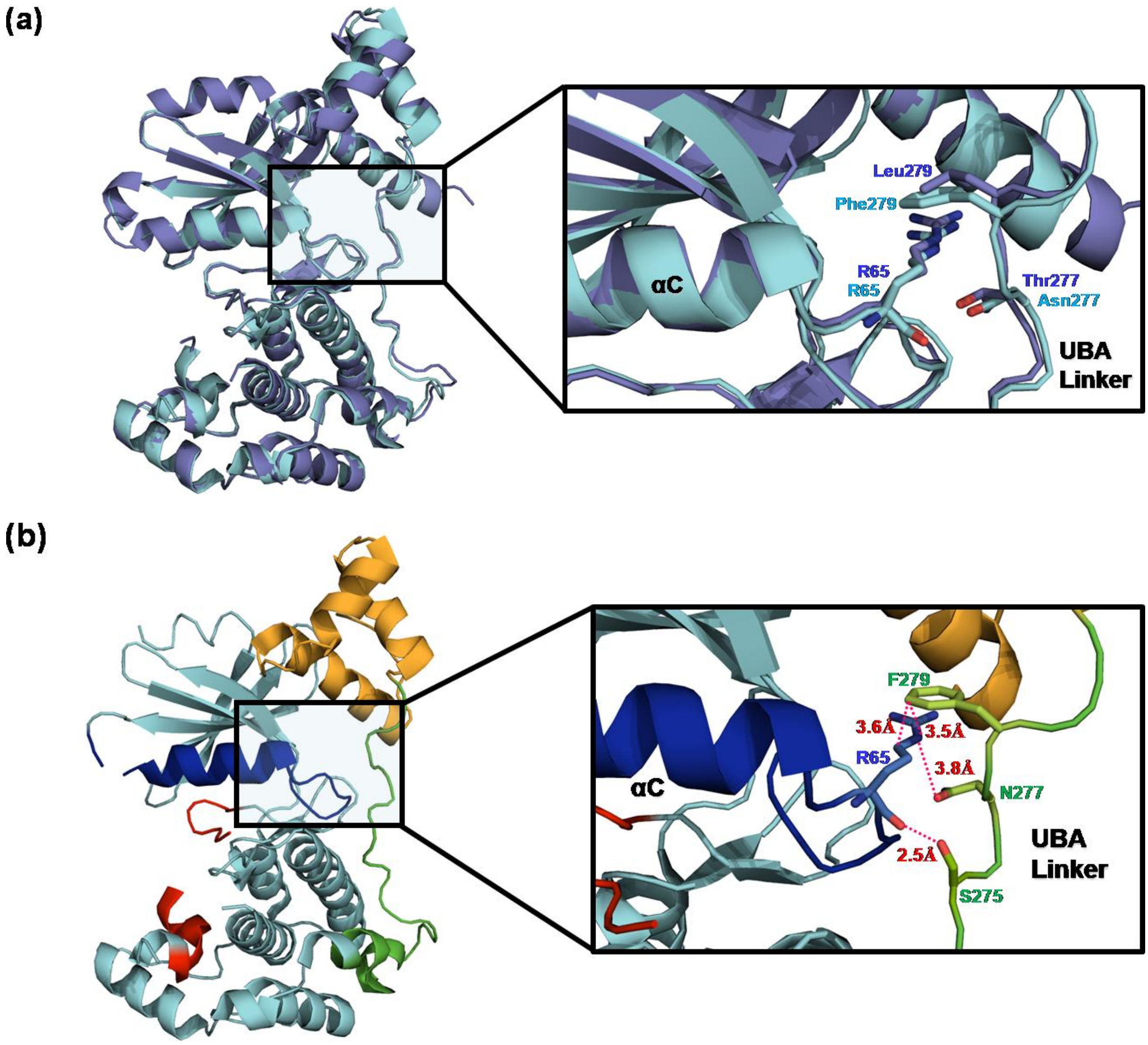

Supporting Figure S5 Superposition of MPK38 and human MELK and the interactions between the UBA linker and the N-lobe in human MELK. (a) MPK38 (blue) and human MELK (cyan) are superimposed and a close-up view of the boxed region are shown. The key residues are depicted as stick. (b) The overall structure of human MELK and a close-up view of the boxed region are shown. The atomic interactions containing the hydrogen bond is displayed as red dashed lines. The distances of the specific interactions are indicated.
